# Bridging the Gap: Early Identification of Mental Health Needs in Paediatric Inpatients

**DOI:** 10.1192/bjo.2025.10676

**Published:** 2025-06-20

**Authors:** Sivakami Sibi, Sophie Marin, Birgit Westphal

**Affiliations:** 1 Queen Mary University of London, London, United Kingdom; 2 Royal London Hospital, London, United Kingdom

## Abstract

**Aims:** Paediatric inpatients often face both physical and mental health challenges, yet the extent of their mental health needs may not always be recognised. At the Royal London Hospital (RLH), we observed a high prevalence of mental health distress among paediatric inpatients, with many scoring above the distress threshold on the Strengths and Difficulties Questionnaire (SDQ), a validated screening tool for emotional and behavioural difficulties. We aimed to assess the prevalence of undiagnosed mental health concerns in paediatric inpatients using the SDQ, hypothesising that 80% would exhibit elevated distress scores, indicating potential unmet mental health needs.

**Methods:** Between 25 November and 22 December 2024, SDQs were administered to all paediatric inpatients across four wards, using parent or self-rated formats (depending on the child’s age and ability). Exclusion criteria included children already receiving mental health support or those not fluent in English or Tamil. Of 62 families approached, 49 (79%) participated, with 43 included in the analysis after excluding incomplete forms. Reasons for declining participation included language barriers (5), fatigue/stress (7), and perceived irrelevance of the study (1).

**Results:** Of the completed SDQs, 74% of children showed elevated scores in one or more categories, with 28% having a high Total Difficulties Score. Parent-reported data identified emotional (39.3%) and peer difficulties (39.5%) as the most prevalent concerns, while self-reports revealed that 59.9% of children reported greater difficulty in prosocial behaviour. Notably, discrepancies were observed in seven children, who reported higher difficulty scores than their parents.

**Conclusion:** The high prevalence of elevated scores across multiple domains suggests that a significant proportion of paediatric inpatients at RLH have unmet mental health needs. Discrepancies between parent and child reports highlight the value of incorporating multiple perspectives in assessments. The proportion of families declining participation underscores barriers to engaging in mental health screening. Routine, systematic screening during admissions could help normalise assessments and improve access to timely support.

